# The role of tumour markers in improving the accuracy of conventional chest X-ray and liver echography in the post-operative detection of thoracic and liver metastases from breast cancer

**DOI:** 10.1054/bjoc.2000.1477

**Published:** 2000-11-22

**Authors:** A Nicolini, A Carpi, P Ferrari, L Anselmi, C Spinelli, M Conte, P Miccoli

**Affiliations:** Department of Internal Medicine, Department of Reproduction and Aging, Department of General Surgery, University of Pisa, Pisa, Italy

**Keywords:** breast cancer, tumour markers, equivocal chest X-ray, equivocal liver echography

## Abstract

The aim of this retrospective study was to assess the value of a serum tumour marker panel in selecting from among the patients with equivocal chest X-ray (CXR) or liver echography (LE) those with thoracic or liver metastases respectively. Between January 1984 and December 1999, 467 (341 non-relapsed and 126 metastatic) breast cancer patients were followed-up postoperatively. Among the 126 metastatic patients 36 showed thoracic (19 patients) or liver (17 patients) metastases, alone or in conjunction with other organs as the first evidence of distant spread. We focused on this series of 377 patients including 341 non-relapsed plus 36 with liver or thoracic metastases. The patients were followed-up after mastectomy with serial determinations of a panel of CEA-TPA-CA15.3 tumour markers, bone scintigraphy, CXR and LE. Up to December 1999, equivocal CXR occurred in 23 (6.1%) patients of whom 11 (47.8%) developed thoracic metastases; 14 (3.7%) patients showed an equivocal LE of whom 5 developed liver metastases. In the 37 patients with equivocal CXR or equivocal LE prolonged clinical and imaging follow-up over 41 ± 36 months (mean ± SD, range 3–163) was used to ascertain the presence or absence of thoracic or liver metastases. In the 23 patients with equivocal CXR the negative and positive predictive values of the tumour marker panel to predict thoracic metastases were 92% and 100% respectively. In the 14 patients with equivocal LE the negative and positive predictive values of the tumour marker panel for prediction of liver metastases were 90% and 100% respectively. This study shows that in breast cancer patients the CEA-TPA-CA15.3 tumour marker panel has a high value for selecting those patients at high risk of developing clinically evident pulmonary or liver metastases from amongst those subjects with equivocal CXR or equivocal LE. © 2000 Cancer Research Campaign http://www.bjcancer.com


*British Journal of Cancer* (2000) **83**(11), 1412–1417
© 2000 Cancer Research Campaign
doi: 10.1054/ bjoc.2000.1477, available online at http://www.idealibrary.com on IDEAL®

http://www.bjcancer.com

### The role of tumour markers in improving the accuracy of conventional chest X-ray and liver echography in the post-operative detection of thoracic and liver metastases from breast cancer

**A Nicolini[1], A Carpi[2], P Ferrari[1], L Anselmi[1], C Spinelli[2], M Conte[2] and P Miccoli[2]**

[1]Department of Internal Medicine, [2]Department of Reproduction and Aging and [3]Department of General Surgery, University of Pisa, Pisa, Italy

Summary The aim of this retrospective study was to assess the value of a serum tumour marker panel in selecting from among the patients with equivocal chest X-ray (CXR) or liver echography (LE) those with thoracic or liver metastases respectively. Between January 1984 and December 1999, 467 (341 non-relapsed and 126 metastatic) breast cancer patients were followed-up postoperatively. Among the 126 metastatic patients 36 showed thoracic (19 patients) or liver (17 patients) metastases, alone or in conjunction with other organs as the first evidence of distant spread. We focused on this series of 377 patients including 341 non-relapsed plus 36 with liver or thoracic metastases. The patients were followed-up after mastectomy with serial determinations of a panel of CEA-TPA-CA15.3 tumour markers, bone scintigraphy, CXR and LE. Up to December 1999, equivocal CXR occurred in 23 (6.1%) patients of whom 11 (47.8%) developed thoracic metastases; 14 (3.7%) patients showed an equivocal LE of whom 5 developed liver metastases. In the 37 patients with equivocal CXR or equivocal LE prolonged clinical and imaging follow-up over 41 ± 36 months (mean ± SD, range 3–163) was used to ascertain the presence or absence of thoracic or liver metastases. In the 23 patients with equivocal CXR the negative and positive predictive values of the tumour marker panel to predict thoracic metastases were 92% and 100% respectively. In the 14 patients with equivocal LE the negative and positive predictive values of the tumour marker panel for prediction of liver metastases were 90% and 100% respectively. This study shows that in breast cancer patients the CEA-TPA-CA15.3 tumour marker panel has a high value for selecting those patients at high risk of developing clinically evident pulmonary or liver metastases from amongst those subjects with equivocal CXR or equivocal LE. © 2000 Cancer Research Campaign http://www.bjcancer.com

**Keywords:** breast cancer; tumour markers; equivocal chest X-ray; equivocal liver echography

The postoperative follow-up strategies for breast cancer patients whether or not they are intensive usually include a relatively large variety of radiological examinations (Winchester et al, 1979; Horton, 1984; Pandya et al, 1985; Muss et al, 1988; Loomer et al, 1991; The GIVIO Investigators, 1994). For detecting bone metastases, skeletal X-rays (Sx-ray), bone scanning (BS), computed tomography (CT) and nuclear magnetic resonance (NMR) may be performed. For chest involvement conventional chest X-ray (CXR) and for liver metastases detection liver echography (LE) and CT are useful.

LE depicts the presence of solid or cystic lesions in the liver. There are no retrospective follow-up studies that have addressed how often LE is the initial examination for detecting liver metastases (ASCO, 1996). Two prospective studies that included LE in an intensive surveillance programme failed to show a survival benefit when compared with minimal surveillance based upon history and physical examination (Rosselli Del Turco et al, 1994; The GIVIO Investigators, 1994).

However, during the postoperative follow-up of breast cancer patients conventional CXR and LE as well as other imaging techniques, are usually performed for prognostic purposes because of demands by the patients or for concomitant transient pathology which needs to be differentiated from distant metastases of breast cancer. Therefore it seems opportune to define the precise indications, sensitivity and specificity of these radiological exams in this setting. Furthermore CXR and LE can give equivocal and false positive results. Recently, the serum concentrations of tumour markers have been used as a warning sign of distant metastases (Neville et al, 1978; Schegel et al, 1981; Nicolini et al, 1989); the combination of carcinoembryonic antigen (CEA), tissue polypeptide antigen (TPA) and breast cancer-associated antigen 115 D8/DF3 (CA15.3) has shown a sensitivity of 87% in the detection of breast cancer relapses with a 78% positive predictive value (Nicolini et al, 1991a).

For many years we have performed a postoperative follow-up programme for breast cancer patients which has included conventional CXR, LE, and the CEA-TPA-CA15.3 tumour marker panel (Nicolini et al, 1991a, 1997, 1999). In a previous retrospective study we have shown that the CEA-TPA-CA15.3 tumour marker panel can increase the accuracy of BS and Sx-ray in diagnosing bony metastases by clarifying equivocal imaging results (Nicolini et al, 1999). The aim of this study was to similarly assess the value of serial determinations of the serum CEA-TPA-CA15.3 tumour marker panel to identify amongst those breast cancer patients with equivocal CXR or LE those affected with or at high risk of developing thoracic or liver metastases respectively.

*Received 19 April 2000*
*Revised 25 July 2000*
*Accepted 9 August 2000*

*Correspondence to*: A Nicolini

### MATERIALS AND METHODS

### Patients

Between January 1984 and December 1999, 467 (341 non-relapsed and 126 metastatic) breast cancer patients were subjected to postoperative follow-up. Among the 126 patients with metastatic disease 36 showed thoracic (19 patients) or liver (17 patients) metastases alone or concomitant with lesions in other organs as the first site of distant spread. We focused on the series of 377 patients including the 341 non-relapsed plus 36 with liver or thoracic metastases. These 377 patients were 29 to 81 years old and had axillary lymph-node involvement (N+) in 139 (36.8%). In one patient, lung metastases were present at the time of mastectomy. The postoperative follow-up visits were carried out every 6 or 4 months depending on whether they were N– or N+ at pathological examination. ER- and PR-patients were allocated to the shorter interval for follow-up. Initially the serum CEA-TPA-CA15.3 panel determination, routine blood tests (ESR, glucose, calcium, phosphorus, blood cell count, BUN, creatinine, GOT, GPT, gamma-GT, bilirubin, alkaline phosphatase, immunoglobulins), conventional CXR, BS, LE and a detailed history and clinical examination were carried out to better define the post-operative staging. Baseline Sx-ray was performed to distinguish benign lesions due to inflammatory and/or degenerative disease during the follow-up. Subsequently, at each postoperative follow-up visit, every 4–6 months, the CEA-TPA-CA15.3 tumour marker panel, the clinical and routine laboratory examinations were performed in addition to the history; BS and LE were usually performed every 24 months and CXR at more prolonged intervals (mean value 42 months). In the 341 non-relapsed patients a total of 686 CXRs and 1364 LE were performed. CXRs were carried out at 56 ± 40 (mean ± SD) and LE at 25 ± 11 (mean ± SD) month intervals. In the 35 relapsed patients the total number of CXRs were 77 and LE were 93. CXRs were performed at 27 ± 29 (mean ± SD) and LE at 19 ± 9 (mean ± SD) month intervals. All the 377 studied patients were followed-up for at least 1 year and the mean time was 98 ± 58 months.

### Tumour markers

TPA was measured by IRMA (Sangtec Medical, Bromma, Sweden) commercial kit. Serum levels initially >60 mU $ml^{-1}$ and subsequently >85 mU $ml^{-1}$ were considered to be elevated. Serum CA 15.3 concentrations were determined by IRMA (Cis International) using a commercial kit and 32 mU $ml^{-1}$ was taken as the cut-off level. CEA was measured initially by Lepetit Lysophase RIA (Milano, Italy) and subsequently by Sorin Biomedica (Saluggia, Italy) commercial kits; both methods gave superimposable results in appropriate comparative studies. Serum levels >7 ng $ml^{-1}$ were considered to be elevated. The within and between assay coefficients of variation for CEA, TPA and CA15.3 were all less than 6% and 9%, respectively. When the TPA cut-off value was 60 mU $ml^{-1}$ the coefficients of variation increased to 10% and 15%, respectively, due to changes in the test procedure using the same method. In our clinical study the serum tumour marker level itself was much less important than their time-related change. A dynamic evaluation of tumour markers was made and in cases of a high tumour marker value a further blood sample was drawn within a month of the previous elevated value. If the remeasured tumour marker value had decreased to a normal level the initial elevated value was considered to be an isolated elevated value. The elevated tumour marker was considered to be progressive when it was 30%, or more, higher in the sample which followed the initial elevated value. Otherwise, two equally high values were regarded to be a constant elevation. Only patients with constant elevation or progressive increase of one or more tumour markers, unexplained by any concomitant benign pathology or history, were considered to be suspicious or indicative of tumour relapse respectively (Nicolini et al, 1991b, 1997).

### CXR and LE and the management of the patients with equivocal finding

All conventional chest X-rays (CXRs) were carried out in 2 projections using the standard techniques. Liver ultrasound was performed in our University Hospital using a 3.5 MHZ transducer with 2 mm resolution. All CXRs and liver ultrasounds were read by skilled radiologists and an official written report was given for each examination.

Conventional CXR and LE were considered equivocal when in the official report lesion(s) were described suspicious for relapse but further examinations were requested to confirm their metastatic origin. The lesions that were considered to be equivocal by conventional CXR were clarified by CT or bronchoscopy and cytologic study. In 2 patients repetitive conventional CXR were soon carried out and prolonged clinical follow-up was used to determine the nature of the equivocal imaging findings. The lesions felt to be equivocal at LE were clarified by LE guided FNA cytology when it was possible. In 1 patient CT and in another one short-term repetition of LE together with prolonged clinical follow-up was used to determine the nature of the equivocal imaging finding.

In all these patients examined a prolonged follow-up (41 ± 36 months, mean ± SD; range 3–163) was used to finally clarify their status whether they were or were not metastatic.

### Statistical analysis

Probably positive tests were defined as (a) CXR and LE that were pathological and (b) tumour marker assay panel with constant elevation or progressive increase in one or more antigens, unexplained by any concomitant transient or chronic benign pathology. CXR and LE showing a picture of equivocal interpretation were also considered to be probably positive tests. A probably positive test which was confirmed by monitoring to death or by the development of a definite clinical-imaging course after the initial result was defined as true positive. Probably negative tests were defined as (a) negative CXR and LE and (b) tumour marker assay panel with normal or isolated elevated value that persisted for longer than 6 months after the first abnormal result. Probably negative tests which were followed by at least 1 year of survival without any clinical-imaging sign of relapse were then evaluated as true negatives.

Sensitivity was defined as TP/(TP+FN)%, specificity as TN/(TN+FP)%, accuracy as TN+TP/(TN+FN+TP+FP), positive predictive value as TP/(TP+FP), negative predictive value as TN/(TN+FN), where FP = false positive, FN = false negative, TP = true positive, TN = true negative.

In patients with CXR or LE with equivocal interpretations the exact Fisher's test was applied to verify whether the tumour

*© 2000 Cancer Research Campaign* 

**Table 1** False positive results for the diagnosis of thoracic or liver metastases in 341 non relapsed breast cancer patients

| Test type | Tn | Result | Probably positive test (*n*) | Test specificity (%) |
|---|---|---|---|---|
| Tumour marker panel | 5682 | 136+ | 0[a] | 100 |
| Chest X-ray | 686 | 12e | 12 | 96.5 |
| Liver echography | 1364 | 9e | 9 | 99 |

Tn = total number of tests; Result = number of patients with (e) equivocal initial imaging result or (+) significantly elevated tumour marker panel; [a]in all 136 patients a transient or chronic benign pathology occurred concomitant with significant elevation (constant and/or progressive) in one or more tumour markers.

**Table 2** False negative results in 19 breast cancer patients with thoracic metastases and in 17 with liver metastases

| Test type | Tn | Result | Probably negative test (*n*) | Test sensitivity (%) |
|---|---|---|---|---|
| Tumour marker panel | 604 | 3– | 2[a] | 94.5 |
| Chest X-ray | 79 | 11e<br>1 negative | 1 | 95[b] |
| Liver echography | 94 | 5e<br>2 negative | 2 | 88[b] |

Tn = total number of tests; Result = number of patients with (e) equivocal initial imaging result or (–) normal or not significantly elevated tumour marker panel; [a]in 2 patients the tumour marker panel remained not significantly elevated for longer than 6 months after the first abnormal results; [b]patients first suspected due to abnormal CEA-TPA-CA15.3 tumour marker panel were included.

marker panel had statistically significant power in distinguishing true positives from true negatives.

### RESULTS

### General outcome and CXR and LE with equivocal interpretation

Up to December 1999, 36 (7.7%) of the 467 patients manifested thoracic (19 patients) or liver (17 patients) metastases as the first site of relapse and in 35 of them these occurred during the post-operative follow-up. 23 of these 35 patients were N+ and the remaining 12 N–. Two patients of the 19 with thoracic relapse and two other subjects of the 17 with metastatic liver involvement are still alive. 89 (23.6%) of the 341 disease-free patients withdrew from follow-up.

During the postoperative follow-up 37 (9.8%) of the 377 patients showed CXRs (23 patients) or LE (14 patients) with equivocal interpretations. 12 of the 23 patients with equivocal CXRs and 9 of the 14 with equivocal LE occurred in the 341 non-relapsed patients. The remaining patients, 11 with equivocal CXRs and 5 with equivocal LE were in the 19 and 17 patients with thoracic and liver metastases respectively.

### Non-relapsed patients: specificity of CEA-TPA-CA15.3 tumour marker panel

Table 1 shows the principal results of CEA-TPA-CA15.3 tumour marker panel, CXR and LE in the 341 non-relapsed patients. In these patients the overall specificity for excluding thoracic or liver metastases of the tumour marker panel, CXR and LE were 100%, 96.5% and 99%, respectively. Importantly, 136 patients showed a significantly elevated tumour marker panel concomitant with a transient or chronic benign pathology. The tumour marker elevation was constant in 81 patients and/or progressive in 55 patients.

Diffuse fat infiltration of liver and/or diabetes, chronic liver failure, hepatic cysts, chronic obstructive bronchopulmonary disease, cholelithiasis, rheumatoid arthritis were the chronic benign pathologies likely responsible for tumour marker constant increase in 42, 9, 3, 2, 2 and 1 patient, respectively. Diffuse fat infiltration of liver and/or diabetes and chronic liver failure also were likely responsible for tumour marker constant and/or progressive increase in 22 and 19 other patients, respectively. Transient liver disorder (indicated by biochemical tests), acute inflammation of joints, upper airways, bladder, lymphatic vessels were the transient benign pathologies likely responsible for tumour marker constant increase in 3, 2, 3, 1 and 1 patient, respectively. Transient liver disorder, acute joint inflammation and acute upper airways inflammation also were likely responsible for tumour marker constant and/or progressive increase in 3, 2 and 1 other patient. Smoking was likely responsible for tumour marker constant increase in 10 patients and for constant and/or progressive increase in 8 other subjects. In 2 patients with tumour marker constant increase no reason likely responsible for tumour marker elevation was found. In most non-relapsed patients with significant tumour marker panel increase the concomitant benign pathology was ascertained by history, physical examination, routine laboratory tests and instrumental investigations which were performed consistent with the protocol. In few of them a well documented history requested by the general practitioner also was available. Therefore no test in addition to those of the protocol was carried out to ascertain the concomitant benign pathology.

12 (3.5%) and 9 (2.6%) of the 341 non-relapsed patients had CXR and LE with equivocal interpretation, respectively. In 18 (86%) of these 21 non-relapsed patients 11 with equivocal CXR and 7 with equivocal LE, CEA-TPA-CA15.3 tumour marker panel was negative. One (8%) of the 12 non-relapsed patients with equivocal CXR showed concomitant constant increase in serum TPA level. This patient was affected by diffuse fat infiltration of liver and diabetes. Two (22%) of the 9 non-relapsed patients with equivocal LE showed concomitant constant increase in serum TPA levels. One of these two patients was affected by diffuse fat infiltration of the liver and diabetes. The other was affected by diffuse fat infiltration of the liver and chronic obstructive bronchopulmonary disease. Therefore none of these 3 patients was considered suspected for relapse. In these 21 patients aged 37–75

*© 2000 Cancer Research Campaign*

**Table 3** Tumour marker panel in 23 patients with an equivocal chest X-ray

| Exam | Non relapsed patients ($n$ = 341) | Patients with thoracic metastases ($n$ = 19) |
|---|---|---|
| Equivocal chest x-ray ($n$) | 12 | 11 |
| Tumour marker panel + ($n$) | 0[a] | 10[a] |
| Tumour marker panel – ($n$) | 12[a] | 1[a] |
| Tumour marker panel specificity | 100% | – |
| Tumour marker panel sensitivity | – | 91% |

(+) significantly elevated; (–) normal or not significantly elevated tumour marker panel; [a]$P$ = 0.00001, exact Fisher's test.

**Table 4** Tumour marker panel in 14 patients with equivocal liver echography

| Exam | Non-relapsed patients ($n$ = 391) | Patients with liver metastases ($n$ = 17) |
|---|---|---|
| Equivocal liver echography (n) | 9 | 5 |
| Tumour marker panel + (n) | 0[a] | 4[a] |
| Tumour marker panel – (n) | 9[a] | 1[a] |
| Tumour marker panel specificity | 100% | – |
| Tumour marker panel sensitivity | – | 80% |

(+) significantly elevated; (–) normal or not significantly elevated tumour marker panel; [a]$P$ = 0.005, exact Fisher's test.

years (53.2 ± 9.5, mean ± SD), 7 (33%) were premenopausal and 5 (24%) were N+ and thoracic and liver metastases were not confirmed during a mean clinical-imaging follow-up over more than 53 months (range 12–163).

### Relapsed patients: sensitivity of CEA-TPA-CA15.3 tumour marker panel

Table 2 shows the principal results of the CEA-TPA-CA15.3 tumour marker panel, CXR and LE in the 36 patients with thoracic (19 patients) or liver metastases (17 patients). In these relapsed patients the overall sensitivity for suspecting thoracic or liver metastases of the tumour marker panel, CXR and LE were 94.5%, 95% and 88%, respectively. Specifically, in 2 patients with thoracic metastases the tumour marker panel remained normal or not significantly elevated for longer than 6 months after the first abnormal result. In a third case with liver metastases a constant increase in serum TPA values occurred 6 months after the first abnormal finding.

In 17 (89%) of the 19 patients with thoracic metastases constant elevation or progressive increase in one or more tumour markers in the panel which was not explained by another concomitant chronic or transient pathology, was the first sign of relapse alone or together with a pathologic (1 patient) (5.2%) or equivocal (5 patients) (26%) CXR. In 7 (63.6%) of the 11 patients with an equivocal CXR thoracic metastases were confirmed by thoracic CT and in 2 others by cytologic study of a sample taken during bronchoscopy. In the 2 remaining cases the progression of the lesions on frequently performed CXRs allowed the metastatic origin of thoracic involvement to be ascertained.

In 16 (94%) of the 17 patients with liver metastases a constant elevation or progressive increase in one or more tumour markers in the panel which could not be explained by an alternative concomitant chronic or transient benign pathology was the first sign of relapse alone (13 patients) or concomitant with equivocal (2 patients) (11.7%) or pathologic (1 patient) (5.9%) LE. In 3 (60%) of the 5 patients with equivocal LE liver metastases were confirmed by LE-guided cytology. In another patient liver metastases were confirmed by liver CT. In the remaining patient the repeated LE finally allowed the metastatic origin of liver involvement to be confirmed.

### The role of tumour markers in confirming or excluding thoracic or liver metastases in the patients with equivocal CXR or equivocal LE

Table 3 shows that among the 23 patients with equivocal conventional CXR only one of the 11 relapsed patients had a normal tumour marker panel or a panel which was not significantly elevated, while all the 12 non-relapsed patients showed a normal (11) or significantly elevated (1) tumour marker panel explained by concomitant benign pathology ($P$ = 0.00001, exact Fisher's test). Therefore, the sensitivity and specificity of the tumour marker panel for detection of thoracic metastases in these patients was 91% and 100%, respectively. Similarly, Table 4 shows that among the 14 patients with equivocal LE only one of the 5 relapsed patients had a normal tumour marker panel or a panel which was not significantly elevated while all the 9 non-relapsed patients had a normal (7) tumour marker panel or a panel which was significantly elevated (2) and explained by concomitant benign pathology ($P$ = 0.005, exact Fisher's test). Therefore sensitivity and specificity of the tumour marker panel for detection of liver metastases was 80% and 100%, respectively.

The positive and negative predictive value of the CEA-TPA-CA15.3 tumour marker panel in the 23 patients with equivocal CXR was 100% and 92%, respectively.

The positive and negative predictive value of the CEA-TPA-CA15.3 tumour marker panel in the 14 patients with equivocal LE was 100% and 90%, respectively.

### DISCUSSION

In breast cancer patients who are or are not submitted to a deliberate postoperative follow-up CXR and LE can be utilized to distinguish secondary thoracic or liver involvement from an alternative pathology. To this purpose conventional CXR and LE

© 2000 Cancer Research Campaign proved to be highly specific examinations (Winchester et al, 1979; Logager et al, 1990; Kruskal and Kane, 1992; The GIVIO Investigators, 1994) but some imaging findings occur which are subjected to equivocal interpretation. Equivocal CXR and LE imaging findings may also occur in the presence of metastatic lesions at the limit of the detectability of these radiological examinations. It is well known that CXR findings can be difficult to interpret to definitely diagnose distant metastases from breast cancer in presence of a single lesion of any size or when multiple nodules show irregular and ill-defined margins. Also the origin of disseminated miliary nodules and of carcinomatous lymphangitis can be difficult to interpret (Fargnoli et al, 1999). With regard to LE the commonest patterns which are difficult to interpret are the following: a) widely ‘irregular’ liver with ‘skip areas’; this picture often occurs in patients previously treated with chemotherapy; b) ‘regular’ liver with single or multiple echogenic areas (Kruskal and Kane, 1992).

When equivocal CXR or LE occur, repetition of the examination in a short time is a simple but not always effective way to solve the problem. Furthermore it entails a delay in starting therapy when the lesions are of metastatic origin. Thoracic or liver CT, or NMR, can usually determine the nature of the non-specific lesions detected by CXR or LE; however they are expensive and time-consuming examinations. In a few instances invasive procedures such as bronchoscopy, LE or CT guided fine needle aspiration (FNA) are necessary to define the nature of the lesions which are equivocal on CXR or LE. Therefore there is a need for simple means to better select patients with equivocal LE or CT for more expensive examinations or invasive procedures.

In this study of breast cancer patients they were monitored postoperatively by means of clinical examinations, routine laboratory tests, CEA-TPA-CA15.3 tumour marker panel and imaging techniques including conventional CXR and LE. Clinical examination, routine laboratory tests and CEA-TPA-CA15.3 tumour marker panel are a tool more easily repeatable, cheaper, more harmless and better accepted by the patients than instrumental examinations. The high accuracy of the tumour marker panel in our study, particularly diagnostic sensitivity in the detection of distant metastases, may partly be due to the high frequency of test repetition compared to that of CXR and LE. However previous studies evaluating the accuracy of intensive follow-up with instrumental examinations for the same purpose including CXR and/or LE did not show accuracy values comparable to those obtained by the tumour marker panel we used (Ciatto and Herd-Smith, 1983; Logager et al, 1990; Schapira and Urban, 1991; The GIVIO Investigators, 1994). The accuracy and negative predictive value of the CEA-TPA-CA15.3 tumour marker panel which we previously reported for diagnosis of bone metastases in patients with equivocal BS (Nicolini et al, 1999) were similar to those we found in this study for diagnosis of chest or liver metastases in patients with equivocal CXR or equivocal LE. However in the previous study on equivocal BS the tumour marker panel showed a lower positive predictive value than in the present study on equivocal CXR and LE (75% vs 100%). It is likely that the more frequent occurrence of equivocal BS in the non-relapsed patients was because of the well known low specificity of this examination (Fogelman, 1991). Equivocal CXR or equivocal LE occur much more rarely in non-relapsed patients.

These data clearly point out that CEA-TPA-CA15.3 tumour marker panel is an accurate tool for selecting patients with equivocal CXR or LE and that only those patients with a concomitant significantly elevated tumour marker panel can be further studied with CT or NMR and/or undergo invasive procedures to further define the nature of lesions. This advantage is limited by the relatively low incidence of equivocal CXR or LE. These results occurred however in 16/36 of our patients with initial thoracic or liver metastases and in 21/341 of the non-relapsed women. The use of tumour markers in the latter patient group (non-relapsed with equivocal CXR or LE) shows the potential advantage for avoiding unnecessary antitumour therapy were such equivocal imaging finding to be interpreted as positive. In this study the CEA-TPA-CA15.3 tumour marker panel alone or together with CXR or LE was the first sign of relapse in 89% and 94% of patients with thoracic and liver metastases respectively. In a recent review of our updated data we confirmed that in some patients the ‘early’ treatment of breast cancer relapses (i.e. when tumour markers are elevated and radiological examinations are not yet positive) significantly prolongs disease-free and overall survival (Nicolini and Carpi, 2000). These important benefits thus improve the cost-effectiveness of postoperative monitoring of the breast cancer patient by using the CEA-TPA-CA15.3 tumour marker panel.

### ACKNOWLEDGEMENT

We thank Professor B Shapiro from the Michigan University, Ann Arbor, Michigan, USA, for careful revision of the manuscript.

### REFERENCES

ASCO Special Article (1996) Clinical practice guidelines for the use of tumor markers in breast and colorectal cancer. *J Clin Oncol* **14**: 2843–2877

Ciatto S and Herd-Smith A (1983) The role of chest x-ray in the follow-up of primary breast cancer. *Tumori* **69**: 151–154

Fargnoli R, Carrieri G and Nizzi Grifi D (1999) Torace: patologia polmonare e pleurica. In: Dal Pozzo G (ed), *Tomografia computerizzata e TC spirale*. Utet: Torino, pp 519–592

Fogelman I (1991) Bone scanning. In: Maisey MN, Britton KE and Gilday DL (eds), *Clinical Nuclear Medicine*, 2nd edn. Chapman and Hall Medical: London, pp 131–157

Horton J (1984) Follow-up of breast cancer patients. *Cancer* **53**: 790–797

Kruskal JB and Kane RA (1992) Correlative imaging of malignant liver tumors. *Semin Ultrasound CT MR* **13**: 336–354

Logager VB, Vestergaard A, Herrstedt J, Thomsen HS, Zedeler K and Dombernowsky P (1990) The limited value of routine chest X-ray in the follow-up of stage II breast cancer. *Eur J Cancer* **26**: 553–555

Loomer L, Brockschmidt JK, Muss HB and Saylor G (1991) Post-operative follow-up of patients with early breast cancer. Patterns of care among clinical oncologists and a review of the literature. *Cancer* **67**: 55–60

Muss HB, McNamara MJ and Connelly RA (1988) Follow-up after stage II breast cancer: A comparative study of relapsed versus non relapsed patients. *Am J Clin Oncol* **11**: 451–455

Neville AM, Patel S, Capp M, Laurence DJ, Cooper EH, Turberville C and Coombes RC (1978) The monitoring role of plasma CEA alone and in association with other tumour markers in colorectal and mammary carcinoma. *Cancer* **42**: 1448–1451

Nicolini A and Carpi A (2000) postoperative follow-up of breast cancer patients: overview and progress in the use of tumor markers. *Tumor Biol* **21**: 235–248

Nicolini A, Carpi A, Di Marco G, Giuliani L, Giordani R and Palla S (1989) A rational postoperative follow-up with carcinoembryonic antigen, tissue polypeptide antigen, and urinary hydroxyproline in breast cancer patients. *Cancer* **63**: 2037–2046

Nicolini A, Colombini C, Luciani L, Carpi A and Giuliani L (1991a) Evaluation of serum CA15.3 determination with CEA and TPA in the post-operative follow-up of breast cancer patients. *Br J Cancer* **64**: 154–158

Nicolini A, Carpi A and Tibaldi C (1991b) The postoperative management of breast cancer patient: new concepts. In Carpi A, Sagripanti A and Mittermayer CH

 *© 2000 Cancer Research Campaign*

(eds): pp 187–203. Sympomed Medical Publisher: Munchen, *Progress in Clinical Oncology*,

Nicolini A, Anselmi L, Michelassi C and Carpi A (1997) Prolonged survival by 'early' salvage treatment of breast cancer patients: a retrospective 6-year study. *Br J Cancer* **76**: 1106–1111

Nicolini A, Ferrari P, Sagripanti A and Carpi A (1999) The role of tumour markers in predicting skeletal metastases in breast cancer patients with equivocal bone scintigraphy. *Br J Cancer* **79**: 1443–1447

Pandya KJ, McFadden ET, Kalish LA, Tormey DC, Taylor SG 4th and Falkson J (1985) A retrospective study of earliest indicators of recurrence in patients on Eastern Cooperative Oncology Group adjuvant chemotherapy trials for breast cancer. A preliminary report. *Cancer* **55**: 202–205

Rosselli Del Turco M, Palli D, Cariddi A, Ciatto S, Pacini P and Distante V (1994) Intensive diagnostic follow-up after treatment of primary breast cancer. A randomized trial. *JAMA* **271**: 1593–1597

Schapira DV and Urban N (1991) A minimalist policy for breast cancer surveillance. *JAMA* **265**: 380–382

Schlegel G, Luthgens M, Eklund G and Bjorklund B (1981) Correlation between activity in breast cancer and CEA, TPA and eighteen common laboratory procedures and the improvement by the combined use of CEA and TPA. *Tumor Diagn* **2**: 6–11

The GIVIO Investigators (1994) Impact of follow-up testing on survival and health-related quality of life in breast cancer patients. *JAMA* **271**: 1587–1592

Winchester DP, Sener SF, Khandeskar JD, Oviedo MA, Cunningham MP, Caprini JA, Burkett FE and Scanlon EF (1979) Symptomatology as an indicator of recurrent or metastatic breast cancer. *Cancer* **43**: 956–960

*© 2000 Cancer Research Campaign*
